# Elevated Procoagulant Endothelial and Tissue Factor Expressing Microparticles in Women with Recurrent Pregnancy Loss

**DOI:** 10.1371/journal.pone.0081407

**Published:** 2013-11-20

**Authors:** Rucha Patil, Kanjaksha Ghosh, Purnima Satoskar, Shrimati Shetty

**Affiliations:** 1 Department of Haemostasis and Thrombosis, National Institute of Immunohaematology (ICMR), KEM Hospital, Parel, Mumbai, India; 2 Nowrosjee Wadia Maternity Hospital, Parel, Mumbai, India; Cordelier Research Center, INSERMU872-Team16, France

## Abstract

**Background:**

15% of reproducing couples suffer from pregnancy loss(PL) and recurs in 2-3%. One of the most frequently hypothesized causes of unexplained PL refers to a defective maternal haemostatic response leading to uteroplacental thrombosis. Hereditary thrombophilia and antiphospholipid antibodies have been extensively described as risk factors for PL in women with unknown aetiology. Recently, a new marker has emerged: the cell-derived procoagulant circulating microparticles(MPs) which have been reported to have a major role in many thrombosis complicated diseases. This study aims to analyze the significance of procoagulant MPs in women suffering from unexplained recurrent pregnancy loss(RPL), and characterize their cellular origin.

**Method and Findings:**

115 women with RPL were analyzed for common thrombophilia markers and different cell derived MPs-total annexinV, platelet(CD41a), endothelial(CD146,CD62e), leukocyte(CD45), erythrocyte(CD235a) and tissue factor(CD142)(TF) expressing MPs and were compared with 20 healthy non-pregnant women. Methodology for MP analysis was standardized by participating in the “Vascular Biology Scientific and Standardization Committee workshop”.

**Results:**

Total annexinV, TF and endothelial MPs were found significantly increased(*p*<0.05, 95% confidence interval) in women with RPL. The procoagulant activity of MPs measured by STA-PPL clotting time assay was found in correspondence with annexinV MP levels, wherein the clot time was shortened in samples with increased MP levels. Differences in platelet, leukocyte and erythrocyte derived MPs were not significant. Thirty seven of 115 women were found to carry any of the acquired or hereditary thrombophilia markers. No significant differences were seen in the MP profile of women with and without thrombophilia marker.

**Conclusion:**

The presence of elevated endothelial, TF and phosphatidylserine expressing MPs at a distance (at least 3 months) from the PL suggests a continued chronic endothelial damage/activation which may get exaggerated at the onset of pregnancy. The data suggests that MPs may contribute to uteroplacental thrombosis and are associated with the pathogenesis of RPL.

## Introduction

Pregnancy itself may be considered a hypercoagulable state wherein changes occur in the blood coagulation system in favor of the procoagulant branch with decreased levels of anticoagulant factors and increased levels of procoagulant factors [1-3]. Pregnancy loss (PL) is the most common complication of pregnancy which affects up to 15% of the reproducing couples and recurs in 2% to 3% of them. Despite a wide range of investigations, no apparent cause can be found in more than 50% of cases [4]. Recurrent pregnancy loss (RPL) is defined as two or more failed pregnancies, wherein the pregnancy is defined as a clinical pregnancy documented by ultrasonography or histopathological test [5]. 

A defective maternal hemostatic response leading to hypoxia secondary to thrombosis of the uteroplacental vasculature has been hypothesized to subsequently lead to the fetal loss. This may include thrombosis in decidual vessels, impairment of trophoblast invasion, villitis and placental microthrombi [6]. Hereditary thrombophilia and antiphospholipid antibodies (aPL) have been extensively described as risk factors for RPL [7]. Recently apart from these thrombophilia markers, a new causative marker in thrombosis complicated conditions has emerged: the cell-derived procoagulant circulating microparticles.

Microparticles (MPs) are submicronic phospholipid vesicles, 0.1 to 1um in size, larger than exosomes (>100nm), derived from different cell types including platelets, endothelial cells, leukocytes and red blood cells besides several other cell types and are found also in normal healthy condition. They are released from cell membranes during activation or apoptosis. Major population of MPs express phosphatidylserine (PS) on their surface endowing them their prothrombotic nature and allowing them to bind to annexin V, and thus used as the main marker to identify and quantitate MPs especially in clinical settings associated with thrombosis. However MPs have also been attributed with various other functions like being pro-inflammatory, pro-angiogenic or immunomodulatory and have been found to have a role in vascular dysfunction [8].

MPs have been found in increased numbers in several prothrombotic conditions like deep vein thrombosis, pulmonary embolism and stroke [9]. There is increasing evidence that MPs are involved in the pathogenesis of RPL. PS exposing MP and endothelial MP levels have been found to be increased in women with recurrent miscarriages [10-12]. Hence, MPs appear as a valuable marker for the detection of *in vivo* cell activation and might have a pathogenic potential in RPL. 

In the present study, we analyzed the role played by PS expressing MPs along with those of platelet, endothelial, leukocyte and erythrocyte origin as well as tissue factor expressing MPs in women suffering from unexplained RPL by using flow cytometry. The association of MPs with the common hereditary and acquired thrombophilia markers was also analyzed. 

## Materials and Methods

### Patients

200 women <40 years of age suffering from RPL (n≥ 2) attending the outpatient department of Obstetrics and Gynaecology of Wadia Maternity Hospital at Mumbai as well as other hospitals were referred to Department of Hemostasis and Thrombosis at National Institute of Immunohaematology, Mumbai for thrombophilia work up between July 2011 to December 2012. RPL was defined as 2 or more losses wherein the pregnancy was documented by an ultrasonography or a histopathological test [5] occurring i) at or before 10^th^ week of gestation-early group ii) beyond 10^th^ week of gestation with or without growth retardation-late group and iii) women with both early and late losses. Clinical features of each patient were recorded and out of these, 115 patients were included in the study only after other presumptive etiological causes of RPL were found to be normal i.e. karyotyping of parents, glucose tolerance test, fasting blood glucose test, hysterosalpingography that excludes any anatomic abnormality, intrauterine adhesions and cervical incompetence and hormonal profile.

### Controls

Twenty healthy women, <40 years of age having at least one live birth and no history of PL, concurrent disease, not on any medication and not currently pregnant were used as controls.

### Ethics Approval

The study was approved by the Institutional Ethics Committee Review Board- “Institutional Committee for Research on Human Subjects, National Institute of Immunohaematology (ICMR)”, written informed consent was obtained from all participants and all investigations were conducted according to the principles expressed in the Declaration of Helsinki.

### Blood Sampling

Blood samples of patients and controls were collected at least 3 months (3 months to 24 months) after last PL or child birth, respectively. Blood was immediately mixed gently with one tenth volume of 0.129 M sodium citrate and then centrifuged at 1500 g for 15 minutes at room temperature twice so as to obtain platelet poor plasma. Plasma was stored at -80°C until use and whole blood was kept for DNA extraction.

### Microparticle Assessment/ Enumeration by Flow Cytometry

Methodology for analysis of MPs has been standardized on Becton, Dickinson and Company (BD) Fluorescence activated cell sorting (FACS) Aria by participating in the “Vascular Biology Scientific and Standardization committee workshop: Standardization of flow cytometry (FCM) – based platelet MPs (PMP) enumeration” [13].

Briefly, 30 µl platelet poor plasma was incubated for 30 minutes at room temperature in the dark with 10µl of annexin V - fluorescein isothiocyanate (FITC) and 15µl of phycoerythrin (PE) labeled specific monoclonal antibody against platelet antigen (CD41-PE, IgG_1_, κ, clone HIP8), activated endothelial antigen (CD 62e-PE, IgG_1_, κ, clone 68-5H11), erythrocyte antigen (CD235a-PE, IgG_2b_, κ, clone GA-R2 (HIR2)), 20µl of PE labeled specific monoclonal antibody against leukocyte antigen (CD45-PE, IgG_1_, κ, clone HI30), endothelial antigen (CD146-PE, IgG_1_, κ, clone P1H12), and TF antigen (CD142-PE, IgG_1_, κ, clone HTF-1). After incubation, samples were diluted in 500 µl of annexin V binding buffer. All the antibodies and buffers were provided by BD Biosciences, United States. Concentration-matched isotype antibodies (IgG1-PE), with no reactivity against human antigens, and FITC-Annexin V in 1) phosphate-buffered saline without calcium and 2) Binding buffer with calcium were used as controls to establish the PE and FITC thresholds. Table **1** lists the monoclonal antibodies used to determine MP levels of specific cells. 

**Table 1:** Monoclonal antibodies used to determine different cell specific microparticle levels.

**Sr no.**	**Microparticle source**	**Monoclonal antibody used**
1.	Total Microparticles	FITC- Annexin V with annexin buffer
2.	Platelets	PE- CD41a
3.	Constitutive Endothelial Cell	PE- CD146
4.	Activated Endothelial Cell	PE- CD62e
5.	Leukocyte	PE- CD45
6.	Erythrocyte	PE- CD235a
7.	Tissue factor expressing cells	PE-CD142

FITC- fluorescein isothiocyanate, PE- phycoerythrin

In order to express MP counts as absolute numbers per micro liter of plasma, 30 ul of counting beads with an established concentration close to 1000 beads/µl (Flow CountTM Fluorospheres; Beckman-Coulter, United States) was added to each sample. 

### Flow cytometric analysis

**Figure 1:**
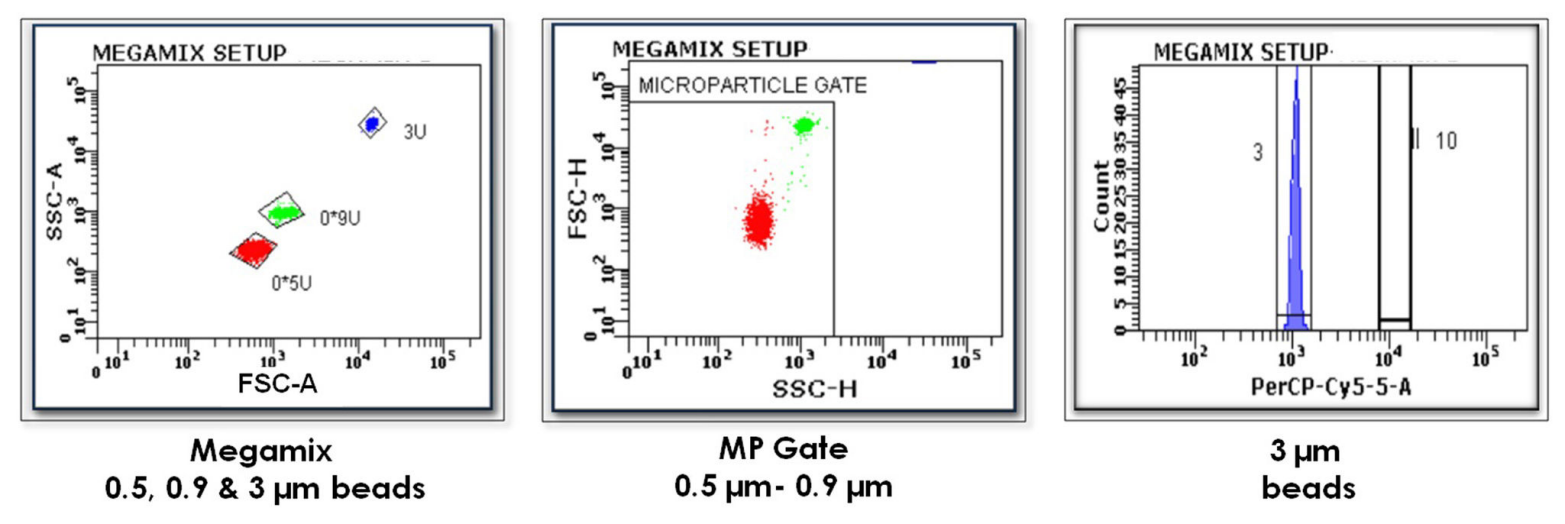
Standardization of microparticle (MP) enumeration. ISTH-“Vascular Biology Workshop”. 1^st^ Graph is a graph of forward scatter (FSC-A) vs. side scatter (SSC-A) parameters showing a blend of monodisperse fluorescent beads (Megamix) of three diameters (0.5, 0.9 and 3 µm). 2^nd^ Graph defines the MP gate which includes all events of 0.9 µm and below (microparticles) and excludes all above (cells). 3^rd^ Graph locates the position of 3 µm beads so as to position the 10 µm counting beads 1 log ahead.

**Figure 2:**
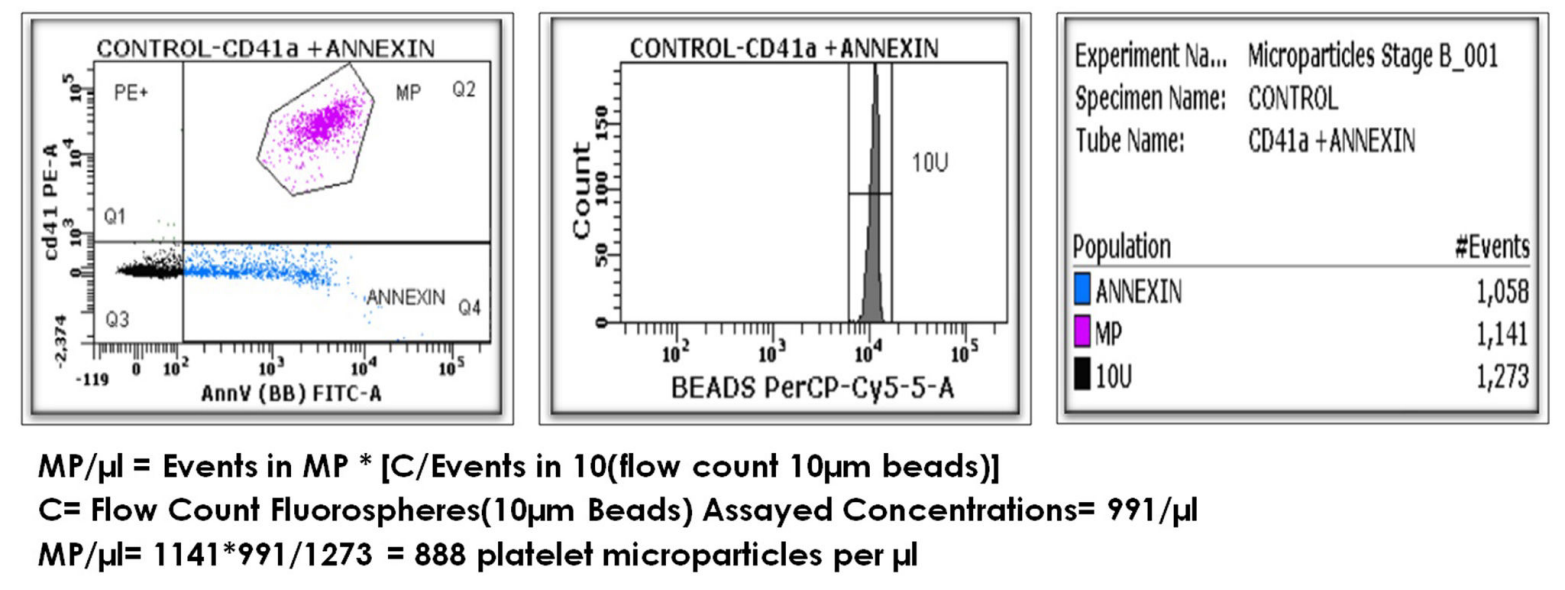
Microparticle(MP) analysis in patients. 1^st^ graph shows dual-positive MPs expressing both FITC- PS and the PE- cell specific marker in quadrant 2 (Q2) and shows MPs expressing only FITC- PS in quadrant 4 (Q4). The total no. of MPs is calculated using the formula: MP/µl = Events in MP * [C/Bead Events (flow count beads)] C= Flow Count Fluorospheres Assayed Concentrations provided by the manufacturer.

Standardization of MP analysis was achieved on BD FACS Aria as shown in Figure **1** using a blend of monodisperse fluorescent beads (Megamix, BioCytex, Marseille, France) of three diameters (0.5, 0.9 and 3 µm). Logarithmic scales for forward scatter (FSC) and side scatter (SSC) parameters were used to cover the wide size range. To check fluorescence compensation settings and to set up positive regions, single stained controls were used. MPs were here defined as dual-positive events expressing both FITC labeled PS and PE labelled cell specific marker seen in dual-color fluorescence plots. The total no. of MPs is calculated using the formula: 

Microparticles/ µl = Events in microparticle gate* [C/ Bead Events (flow count beads)] C= Flow Count Fluorospheres Assayed Concentrations provided by the manufacturer as shown in Figure **2**.

### MP detection by the STA-Procoag-PPL clotting time assay

In order to cross verify the results obtained by flow cytometry and to check the procoagulant activity of MPs, STA- Procoag-PPL clotting time was done in all control samples and in some patient samples found to have increased PS exposing MP levels by flow cytometry.

The STA-Procoag-PPL assay (Diagnostica Stago, France) is a method by which the procoagulant activity of phospholipid i.e. PS expressing MPs can be assessed by measuring the phospholipid-dependent clotting time. The principle of this method is that samples containing elevated levels of phospholipid exposing MPs will shorten the activated FX clotting time. Phospholipid depleted plasma- reagent 1, provides all the coagulation factors and reagent 2 activates FX resulting in thrombin generation.

### Detection of common hereditary thrombophilia markers

Protein C and protein S levels in the patient’s plasma were measured by Enzyme linked Immunosorbent assay. Antithrombin levels were detected by a chromogenic substrate method in fully automated coagulometer using commercial kits (Diagnostica Stago, Asnieres, France); factor V Leiden mutation by allele specific polymerase chain reaction and Methylenetetrahydrofolate reductase (MTHFR C677T) polymorphism by restriction fragment length polymorphism method. 

### Antiphospholipid antibodies analysis

#### Lupus Anticoagulant

Lupus anticoagulant was diagnosed according to the International Society of Thrombosis and Haemostasis (ISTH) Subcommittee recommendations [14] using commercial reagents (Siemens, Munich, Germany).

#### Anti-cardiolipin, Anti-β2 Glycoprotein I, Anti-annexin V Antibodies

These antibodies, both IgG and IgM, were assayed using commercial enzyme linked immunosorbent assay kits. (Generic Assays, Berlin, Germany)

### Statistical Analysis

Increased level of MPs was defined as level > 2 standard deviations (SD) from the mean of control group. MP levels were also compared for the different groups versus controls using the Student’s t-test. Statistical significance was assumed at P < 0.05, 95% confidence interval (CI). Data was analyzed using SPSS statistical software.

## Results

### Characteristics of Patients

115 women belonging to RPL group and 20 healthy women were analyzed for the common hereditary thrombophilia markers, aPL and circulating MP levels of different cell origin. 78 of these 115 patients suffered from ≥ 3 RPL and 37 from 2 RPL and as a debate still exists on the definition of RPL, the analysis of results were done by considering them as 2 separate groups. The median number of consecutive RPL was 3.3 ranging from 2-11. There were in total 3 secondary aborters (recurrent losses after one live birth). Of the 78 cases (group A), 35 (44.8%) experienced early RPL before 10^th^ week of gestation (group B), 16 (20.5%) experienced late RPL, after 10^th^ week of gestation (group C) and 27 (34.6%) experienced both early and late losses (group D). Of the 37 cases (group I), 18 (48.6%) experienced early RPL before 10^th^ week of gestation (group II), 10 (27%) experienced late RPL, after 10^th^ week of gestation (group III) and 9 (24.3%) experienced both early and late losses (group IV). 

### Acquired and hereditary thrombophilia

Among the hereditary thrombophilia, 7 had reduced protein C levels, 10 had reduced protein S, 5 had reduced antithrombin levels, 3 were factor V Leiden heterozygotes, 3 were homozygous for MTHFR and in total 22 patients had at least one genetic marker. Among the different aPL, 9 were positive for lupus anticoagulant, 5 for anti-cardiolipin antibodies, 3 for anti-β2Glycoprotein I antibodies, 10 for annexin V antibodies and in total, 20 patients had one or more acquired thrombophilia markers. Table **2** shows the prevalence of all the thrombophilia markers in these women. In all, thirty seven women were found positive for one or more hereditary or acquired thrombophilia markers analyzed.

**Table 2:** Prevalence of hereditary thrombophilia markers and antiphospholipid antibodies in women with recurrent pregnancy loss.

**Hereditary Thrombophilia and Antiphospholipid antibodies**	**Women with ≥ 3 RPL (n=78)**	**Women with ≥ 2 RPL (n=37)**	**Total no. of women with RPL (n=115)**
**Lupus anticoagulant**	6	3	9
**Anti Cardiolipin Ab**	3	2	5
**Anti β2GP Ab**	2	1	3
**Anti Annexin Ab**	7	3	10
**Protein C deficiency**	7	0	7
**Protein S deficiency**	7	3	10
**AT deficiency**	3	2	5
**FVL heterozygous**	3	0	3
**MTHFR homozygous**	1	2	3

RPL= recurrent pregnancy loss, Ab= Antibody, β2GP= β2 glycoprotein, AT= Antithrombin, FVL= Factor V Leiden, MTHFR= Methylenetetrahydrofolate reductase

### Circulating Microparticles

#### A: Flow cytometry

**Figure 3:**
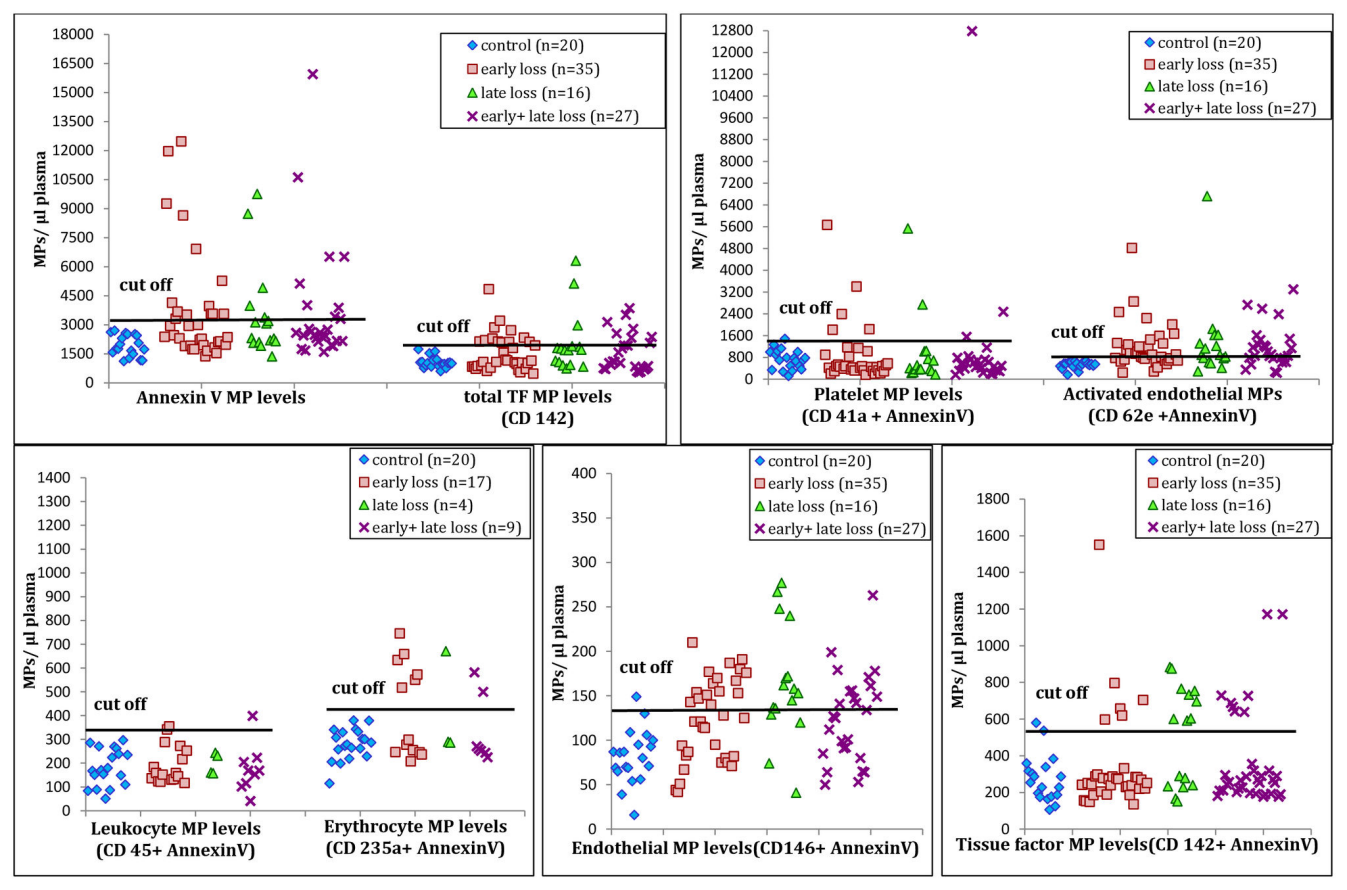
Cell derived microparticle levels in women suffering from ≥ 3 recurrent pregnancy loss when compared to controls. Scatter plots of MP levels of different cell types in different groups of patients along with controls are shown in the 5 graphs. Levels >2 SD from the mean of control women are considered as cut-offs to have increased MP levels.

**Figure 4:**
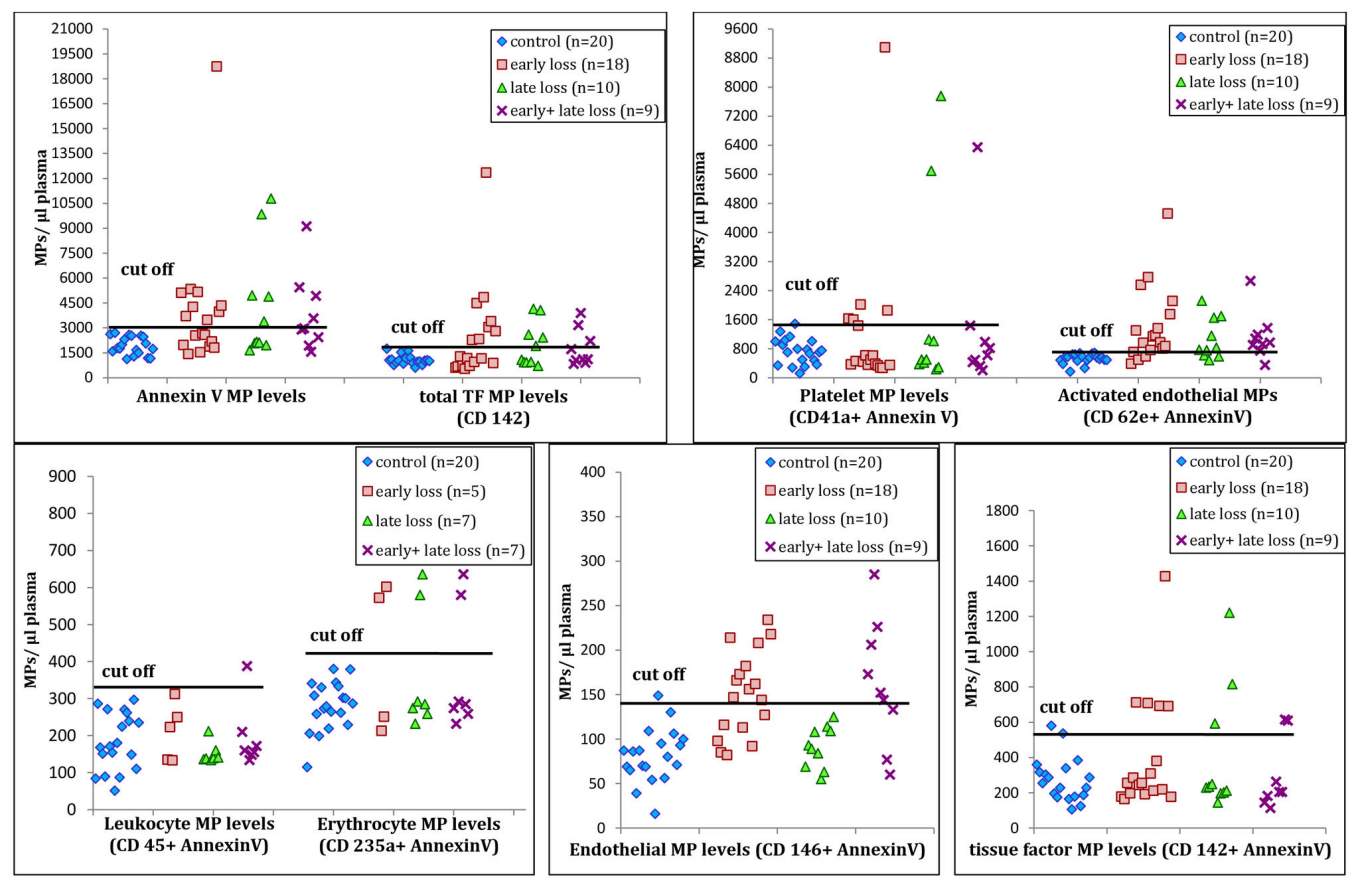
Cell derived microparticle levels in patients suffering from 2 recurrent pregnancy loss when compared to controls. Scatter plots of MP levels of different cell types in different groups of patients along with controls are shown in the 5 graphs. Levels >2 SD from the mean of control women are considered as cut-offs to have increased MP levels.

MP levels of different cell types were determined in 115 patients and 20 controls. Levels >2 SD from the mean of control women were considered as cut-off to have increased MP levels. The scatter plots of MPs of different cell types in patients along with controls are shown in Figure **3** and Figure **4** for all groups i.e. early losses, late losses and both early and late. No differences were observed in the MP profile of women with ≥3 RPL and 2 unexplained RPL. Total procoagulant annexin V MPs, TF expressing MPs and endothelial MPs, both constitutive and activated, were found significantly increased in patients belonging to all groups when compared to the control group (p <0.05, 95% CI). Differences in platelet (CD41a), leukocyte (CD45) and erythrocyte (CD235a) MP levels were not found to be significant.

#### B: STA-Procoag-PPL clotting time

The procoagulant phospholipid-dependent clotting time was found to be proportionately shortened in patients who were found to have increased PS expressing MPs when compared to controls. The Mean± SD values of the clotting time (secs) and the corresponding annexin positive MP levels are shown in Table **3**.

**Table 3:** Procoagulant activity assessment: STA PPL clot time in controls and patients with increased MP levels.

**Sr no.**	**Subjects**	**STA-PPL Clot time (secs) Mean ± SD**	**Annexin V MPs/ µl plasma Mean ± SD**
1	**Controls (n= 20)**	71.9± 6.3	1916.8± 544.8
2	**Patients (n=25)**	44.7± 13.5	8905.5± 5443.2
2a	Very high MPs (n=6)	25.1± 9.2	15085.5± 6961.9
2b	Moderately increased MPs (n=10)	49.8± 3.3	8329± 1440.1
2c	Mildly increased MPs (n=9)	54.4± 2.6	4847.1± 502.5

MP- microparticle, SD- standard deviation, tTF- total tissue factor

### Hereditary thrombophilia Markers, Antiphospholipid antibodies and Circulating Microparticles

The MP levels of the 20 women, who were positive for one or more anti phospholipid antibodies, and the 22 women with at least one hereditary thrombophilia marker were compared with MP levels of the remaining patients who were negative for any marker or antibody. No significant differences were seen in the MP profile of women with or without any thrombophilia marker (*p*> 0.05). The mean ± SD of MP levels of these patient groups are given in Table **4**.

**Table 4:** Comparison of microparticle levels in women with and without thrombophilia marker.

	**Patients without any thrombophilia marker (n=78)**	**Women positive for at least one antiphospholipid antibody (n=20)**	**Women with at least one hereditary thrombophilia marker (n=22)**
**MP Type**	**Mean± SD (MP/ µl)**	**Mean± SD (MP/ µl)**	**Mean± SD (MP/ µl)**
**Total Annexin V**	4216.4± 4883.7	3115.6 ± 2100	3457.3 ± 2025.1
**Platelet CD41a+ Ann**	1454.2±3937.2	801.8 ± 1661.4	990.4 ± 1208.9
**Endothelial CD 62e+ Ann**	1293.9±1040.7	901.3 ± 446.9	1165.5 ± 720.7
**Endothelial CD146+ Ann**	138.9± 53.4	112.2 ± 58.5	130 ± 52.4
**Leukocyte CD45+ Ann**	349.7± 163.9	354.8 ± 126.6	318.1 ± 165.9
**Erythrocyte CD235+ Ann**	636.1± 199.4	501.5 ± 157	500.3 ± 194.9
**Procoagulant TF CD142+ Ann**	547.4± 321.3	476.1 ± 310.3	413.9 ± 175.3
**Total TF CD142**	2338.8± 3052.7	1305.8 ± 916.2	1155.5 ± 922.7

MP- Microparticle, TF- Tissue factor, SD- standard deviation, Ann- Annexin V

## Discussion

Unexplained PL and RPL are often associated with uteroplacental thrombosis and the classical thrombophilia markers account for a certain fraction of these patients [7,15]. However a large percentage of these women show the absence of these markers. Therefore, this study has been undertaken to see whether cell derived MPs may have a role to play in these unexplained cases. Our findings showed that a large number of women with RPL have increased MP levels, mainly PS and TF expressing and endothelial derived MPs, which questions their possible clinical relevance. The procoagulant activity measured by STA-PPL clot based assay not only corresponds to the data obtained by the flow cytometry method but also signifies the procoagulant nature of the MPs. 

Both PS and TF promote and initiate the clotting cascade. MPs also express PS and TF on their surface and thus may contribute in initiating the assembly of different clotting complexes developing a prothrombotic condition. Also due to their small size, MPs may escape the elimination system, remaining longer in circulation than activated or apoptotic cells dispersing their prothrombotic potential in microcirculation. 

Circulating MPs have been associated with different thrombosis complicated clinical [16], acute coronary syndromes [17], chronic renal failure [18] and recently these MPs seem to be a marker or causative agent of different bad obstetric outcome, mainly preeclampsia [19,20] and RPL [10-12,21].

Our study is different from those of others previously reported on association of MPs with RPL for various reasons. All the samples were processed uniformly with regard to sample collection, time spent between sample collection and processing, the sample processing and time period for storage at -80°C. Second, we have analyzed not only PS expressing, platelet and endothelial MPs, but also studied leukocyte, erythrocyte and more importantly TF expressing MPs. No correlation however, was observed between the MP levels and number of pregnancy losses in these women. We found significant increase in PS and TF expressing and endothelial, both activated and constitutive, MP levels in women suffering from PL- early and/or late losses. These findings are similar to those of Laude et al [10] who showed that the MP’s prothrombotic activity is much higher in women with RPL when compared to non- pregnant healthy controls using prothrombinase assay by capture of MPs on immobilized annexin V. Carp et al [11] also showed that endothelial MPs are increased in women with recurrent miscarriage which is contradictory to the report by Alijotas Reig et al [12] who have shown a significant decrease in endothelial MPs in whole group of PL as also with recurrent miscarriage group. In this study, however, the blood samples were collected at the time of diagnosis of the loss and the MP profile was compared to non-matched healthy pregnant controls. It is important to note that the increased MP levels found in our study in patients when compared to controls, as well as in the study done by Laude et al and Carp et al [10,11] were detected at a distance from loss or delivery, respectively (at least 3 months). At least 3 months of time period after the loss for patients or delivery for controls was chosen as haemostatic changes noted during pregnancy normalizes after delivery within 4 to 6 weeks. Platelet count and protein S levels take a little longer [22] and same would be the case after PL. Also on comparing the MP levels in patients and controls whose blood samples were collected 3 months to 6 months after loss/ delivery with those collected 6 months to 24 months after, no difference in MP profile was seen suggesting that 3 months period is sufficient for MP levels to normalize and in these normalized conditions the MP levels were found increased in patients when compared to controls. The aim was to see if MPs were increased in these patients in normalized conditions suggesting a chronic but asymptomatic state of activation and damage. Increase in TF and endothelial MPs may suggest a continued chronic endothelial damage or activation and this may contribute during pregnancy to placental dysfunction and subsequent loss. Also the presence of endothelial MPs in the interval between pregnancies may be a chronic state of blood vessel activation which only becomes apparent in pregnancy. None of the patients in this study had any thrombotic episode. Thus, the presence of elevated MPs in these women may reflect an ongoing systemic pathological yet asymptomatic status, which can turn deleterious in the setting of pregnancy. This may explain the results in the study done by Alijotas Reig et al [12]. As suggested by the authors, the decrease in MP levels may be due to their consumption through excessive clotting initiation and activation in the placental beds and thus they would be trapped in fibrin deposits. Another study has shown that injection of PS containing phospholipid vesicles to pregnant mice induces significant reduction of fetal weight and the placental tissue revealed severe congestion with fibrin depositions. This reduction in fetal weight was inhibited in mice injected with recombinant annexin V [23]. Annexin V is endogenously located mainly at the apical surface of sycytiotrophoblasts which shows high affinity for anionic phospholipids and has an important role in the maintenance of blood flow through the placenta and thus is important for the fetal growth and viability [24]. In women with aPL and a history of PL, a reduced expression of annexin V with enhanced thrombin formation on trophoblasts has been reported by Rand et al [25]. Thus placental annexin V could be one of the targets for PS expressing MPs in pregnancy which may lead to placental thrombosis and this fetal loss. In addition to their prothrombotic nature, they are also known for their pro-inflammatory and pro- apoptotic attributes [8] which may interfere in successful implantation and growth of the embryo. Also pregnancy itself is a hypercoagulable state and the presence of increased MPs may just lead to an exaggerated hemostatic response leading to thrombosis of the uteroplacental vasculature and subsequent fetal loss. 

### Conclusion

From the data obtained we may come to the following conclusions: increased MP levels in women with RPL at a distance from their loss reflect an ongoing state of activation and damage which may become apparent, symptomatic and exaggerated at the onset of pregnancy. 

Thus in the search for the underlying cause of unexplained RPL, the study and detection of circulating MPs seems to be a promising approach. The current study clearly shows the presence of elevated procoagulant MPs in the peripheral circulation of women with early and/or late unexplained pregnancy loss, thus adding to the new emerging body of evidence that MPs have a significant role to play in thrombosis complicated conditions. The question that arises is that if these MPs are associated to PL by developing or assisting in developing a prothrombotic state, will anticoagulants, which have been reported to have a beneficial effect in antiphospholipid antibody syndrome as well as hereditary thrombophilia [26] help prevent subsequent thrombosis, and thus subsequent PL in these patients with increased levels of procoagulant MPs. The effect of anticoagulants and steroids on MP levels should be studied. All these studies could thus open new areas of investigation for the understanding, follow up and therapeutic handling of unexplained pregnancy losses.
